# Enhancing Sensing Performance of Capacitive Sensors Using Kirigami Structures

**DOI:** 10.3390/s24216930

**Published:** 2024-10-29

**Authors:** Chor-Kheng Lim

**Affiliations:** Department of Art and Design, YuanZe University, No. 135, Yuandong Rd., Zhongli Dist., Taoyuan City 320315, Taiwan; kheng@saturn.yzu.edu.tw

**Keywords:** capacitive sensors, Kirigami structures, sensing performance, edge effects, smart home applications

## Abstract

Capacitive sensors have widespread applications in human-machine interaction, Internet of Things, and smart home systems due to their low cost, high sensitivity, and ease of integration. However, improving the sensitivity and sensing distance of capacitive sensors remains a challenging issue. This study proposes a novel capacitive sensor design method based on Kirigami structures, which enhances sensor performance by introducing specific cutting patterns into the conductive layer to leverage edge effects. Through experimental testing and statistical analysis, we systematically investigated the influence of Kirigami geometric parameters on sensor sensitivity and sensing distance. We designed and fabricated 12 different Kirigami structures, including circular flower patterns, array patterns, layered pointed flower patterns, and circular strip structures, and compared them with traditional non-cut structures. The results show that Kirigami structures significantly improved sensor performance. Compared to traditional sensors without Kirigami structures, optimally designed Kirigami capacitive sensors demonstrated approximately a 3-fold increase in sensitivity and up to 170 percent extension in sensing distance. Multivariate regression analysis and nonlinear models revealed complex relationships between Kirigami structural parameters and sensor performance. Notably, the circular strip (three-layer) structure exhibited the best performance, possibly due to its maximization of edge effects and optimization of electric field distribution. This study provides new design insights for developing high-performance capacitive sensors, with potential applications in improving smart home systems and indoor activity monitoring for solitary elderly individuals.

## 1. Introduction

### 1.1. Research Background

Capacitive sensing technology has become increasingly important in modern applications, ranging from human–machine interfaces to smart home systems. Despite its widespread use, traditional capacitive sensors face significant challenges, particularly in non-contact sensing scenarios and complex behavioral pattern recognition. This study addresses these limitations by proposing a novel approach: the integration of Kirigami structures into capacitive sensor design. Our research aims to enhance sensor performance, specifically focusing on improving sensitivity and extending sensing distance, to meet the growing demands of advanced sensing applications.

Capacitive sensors play a crucial role in modern technology, with applications spanning human–machine interaction, Internet of Things, and intelligent systems [1,2]. These sensors offer advantages such as low cost, high sensitivity, and ease of integration [3]. However, as application scenarios become increasingly complex, the demands on sensor performance continue to rise, particularly in enhancing sensitivity and expanding sensing distance [4,5].

However, as the applications of capacitive sensors become more sophisticated, several limitations of traditional designs have become apparent. These include the following:

- Difficulty in distinguishing complex behavioral patterns.
- Inaccuracies in measuring object distance, particularly at longer ranges.
- Rapid signal attenuation as sensing distance increases.
- Insufficient resolution for detecting subtle behavioral differences.

These challenges significantly restrict the application of conventional capacitive sensors in dynamic environments and complex behavioral monitoring scenarios, highlighting the need for innovative solutions in sensor design.

The working principle of capacitive sensors is based on changes in capacitance between electrodes. When a conductor (such as the human body) enters the sensing distance, it alters the electric field distribution between the electrodes, thereby causing a change in capacitance [6]. However, traditional planar electrode structures have significant limitations in non-contact sensing, especially in distinguishing complex behavioral patterns and accurately measuring object distance [7,8].

The author’s previous research [9] developed a proximity sensing module called Presence Sticker, which utilizes capacitive sensing to detect human behaviors such as “passing by”, “staying”, and “touching” in home environments. Presence Sticker highlighted challenges in meeting sensing requirements across various distances and directions. Specifically, it revealed that signal strength attenuates rapidly as sensing distance increases, resulting in reduced accuracy. Additionally, the system struggled to distinguish subtle behavioral differences with sufficient resolution. The present study builds upon these findings, aiming to optimize sensor sensitivity and address the challenges identified in the previous research.

### 1.2. Principles and Development of Capacitive Sensing Technology

Capacitive sensing technology operates on the principle of detecting changes in capacitance between electrodes to sense the presence, position, or other physical characteristics of objects. In their comprehensive review, Wang et al. [1] highlighted that the performance of capacitive sensors is primarily influenced by electrode design, dielectric material selection, and readout circuits. In recent years, capacitive sensing technology has achieved significant advancements across multiple domains:

- Material Innovation: Zang et al. [2] conducted a thorough review of the development of flexible pressure sensors, emphasizing the crucial role of novel conductive materials in enhancing sensor sensitivity.
- Structural Optimization: Boutry et al. [5] introduced an innovative biomimetic electronic skin with a hierarchical structure, demonstrating the potential of bio-inspired structural designs in improving sensor functionality.
- Signal Processing: The study by Gu et al. [4] emphasized the significance of advanced signal processing techniques in enhancing the performance of capacitive sensors, particularly in mitigating environmental interference and extending sensing distance.

### 1.3. Challenges Faced by Capacitive Sensors

Despite these advancements, capacitive sensors still face several technical challenges:

- Sensing Distance Limitation: Wang et al. [6] demonstrated that traditional planar electrode structures suffer from signal attenuation issues in long-distance sensing.
- Environmental Interference: Boutry et al. [5] highlighted the significant impact of environmental factors such as humidity and temperature on sensor accuracy.
- Spatial Resolution: Xu et al. [7] emphasized the need for enhancing spatial resolution in non-contact scenarios.
- Multi-Functional Integration: Chortos et al. [3] stressed the challenges in integrating multiple sensing functions into a single sensor.

These challenges underscore the need for innovative approaches in capacitive sensor design, particularly for applications in smart homes and elderly care domains.

In light of these challenges, there is a pressing need for innovative approaches to enhance the performance of capacitive sensors. One such promising solution lies in the integration of Kirigami structures into sensor design. Kirigami, an ancient Japanese paper-cutting art, has recently gained attention in the scientific community for its potential to impart unique mechanical and functional properties to materials through specific cutting patterns. The application of Kirigami principles to capacitive sensor design offers a novel pathway to address the current limitations and push the boundaries of sensor performance.

### 1.4. Potential of Kirigami Structures

To address the aforementioned challenges, this study proposes the application of Kirigami structures in capacitive sensor design. Kirigami, originating from Japanese paper-cutting art, can impart unique mechanical and functional properties to materials by introducing specific cutting patterns [10]. In recent years, Kirigami structures have garnered widespread attention in materials science and flexible electronics, with research demonstrating their ability to enhance material flexibility, extensibility, and multi-functionality significantly [11,12]. In the field of electronics, Kirigami structures have been successfully applied to develop high-performance flexible sensors, wearable devices, and flexible displays [13,14]. For instance, Xu et al. [15] developed a highly stretchable micro-supercapacitor using Kirigami structures, which maintained stable electrical and electrochemical performance under extreme deformation. More recently, Kim et al. [16] further demonstrated the potential of Kirigami structures in electrode design, developing Kirigami-structured electrodes that not only exhibited excellent stretchability (up to 350 percent tensile strain) but also maintained low resistance (7.6 Ω/sq) and stable electrical performance. By applying Kirigami structures to capacitive sensor design, we anticipate several key benefits:

- Enhanced Edge Effects: The intricate patterns of Kirigami structures can significantly increase the effective edge length of electrodes, potentially enhancing the sensor’s sensitivity to small changes in capacitance.
- Optimized Electric Field Distribution: The unique geometric shapes created by Kirigami cuts may lead to more complex and beneficial electric field distributions, possibly improving spatial resolution and sensing distance.
- Improved Flexibility and Adaptability: Kirigami-structured sensors may maintain stable performance under various deformation conditions, crucial for applications in smart homes and medical monitoring where sensors need to adapt to complex environments.

This structural optimization may not only increase sensor sensitivity but also enable sensors to maintain stable performance under various deformation conditions, which is particularly important for smart home and medical monitoring applications that need to adapt to complex environments. Our approach aims to address the current limitations of traditional capacitive sensors while opening new possibilities for their application in more demanding scenarios, such as precise behavior recognition and long-range sensing in smart home environments.

### 1.5. Research Hypotheses and Scientific Basis

Based on the unique characteristics of Kirigami structures, this study proposes two main hypotheses:

1. Edge Effect Enhancement Hypothesis: Applying Kirigami structures to capacitive sensor design can significantly increase the effective edge length of electrodes, thereby enhancing edge effects and improving sensor sensitivity.

2. Electric Field Distribution Optimization Hypothesis: The special geometric shapes of Kirigami structures can create more complex and specific electric field distributions, thus improving sensor performance, especially in terms of sensing distance.

The combination of these two hypotheses is expected to significantly enhance the overall performance of capacitive sensors, including sensitivity and sensing distance. These hypotheses are based on the following scientific foundations:

1. Edge Effect Enhancement: Kirigami structures can significantly increase the effective edge length of electrodes. According to capacitive sensing theory, the electric field strength is higher at the edges of electrodes, contributing more to capacitance changes [17].

By increasing edge length, the sensor’s sensitivity to small changes can be improved.

2. Electric Field Distribution Optimization: The special geometric shapes of Kirigami structures can alter the electric field distribution around the electrodes. Studies have shown that non-uniform electric field distributions can improve the spatial resolution of sensors [18]. Through carefully designed Kirigami patterns, we expect to create more complex and specific electric field distributions, thereby improving sensor performance, especially in extending sensing distance.

### 1.6. Research Problems

Based on the limitations of traditional capacitive sensors and the potential of Kirigami structures, this study addresses the following key research problems:

- How can Kirigami structures be effectively integrated into capacitive sensor design to enhance edge effects and optimize electric field distribution?
- What is the quantitative relationship between Kirigami structural parameters (such as cutting pattern complexity and edge length) and sensor performance metrics (sensitivity and sensing distance)?
- How do different Kirigami patterns affect the sensor’s ability to distinguish complex behavioral patterns and maintain accuracy over extended sensing distances?
- Can Kirigami-structured capacitive sensors provide more stable and reliable performance in dynamic and deformable environments than traditional planar sensors?
- What are the optimal Kirigami designs for maximizing both sensitivity and sensing distance in capacitive sensors?

Addressing these research problems will advance our understanding of Kirigami-enhanced capacitive sensing and provide practical solutions for developing next-generation sensors with superior performance characteristics.

### 1.7. Research Objectives

These research problems highlight the multifaceted challenges in applying Kirigami structures to capacitive sensor design. Addressing these issues requires a systematic approach that combines theoretical analysis, experimental design, and practical validation. We have formulated the following research objectives to tackle these challenges and advance our understanding of Kirigami-enhanced capacitive sensors. These objectives are directly aligned with the identified problems and aim to provide comprehensive solutions that bridge the gap between Kirigami structure design and capacitive sensor performance enhancement:

1. Design and fabricate Kirigami-based capacitive sensor: Develop a new sensor prototype that enhances edge effects to improve sensing sensitivity while maintaining the original sensor area through Kirigami structures.

2. Systematically study the influence of Kirigami structural parameters on sensor performance: Investigate how geometric parameters such as cutting density and shape affect sensor sensitivity and sensing distance. This will involve detailed parameter scanning experiments and performance testing.

3. Establish mathematical models: Develop mathematical models describing the relationship between Kirigami structures and capacitive sensing performance. This will include multivariate regression analysis of experimental data and exploration of nonlinear relationships to establish predictive models for structure–performance relationships.

4. Performance comparison: Compare the performance differences between Kirigami structure-improved sensors and traditional planar electrode sensors used in previous studies under controlled laboratory conditions. Focus on evaluating sensitivity improvement and sensing distance extension.

5. Explore practical application potential: Based on experimental results, assess the application possibilities of Kirigami structure capacitive sensors in actual scenarios such as smart home systems and indoor activity monitoring for solitary elderly individuals.

## 2. Kirigami Structures: Past, Present, and Potential in Sensor Design

This session delves into the fascinating world of Kirigami structures, exploring their origins, fundamental principles, and emerging applications in materials science and sensor design. We begin by examining the basic concepts underlying Kirigami and its transformation from an ancient art form to a cutting-edge tool in modern engineering. The chapter then progresses to discuss the diverse applications of Kirigami in materials science, with a particular focus on nanomaterials and electronic components. Finally, we explore the immense potential of Kirigami structures in sensor design, highlighting current research gaps and future opportunities. This comprehensive overview aims to provide a solid foundation for understanding the role of Kirigami in advancing capacitive sensor technology, setting the stage for the innovative approach proposed in this study.

### 2.1. Fundamental Principles of Kirigami Structures

Kirigami, derived from the Japanese words “kiri” (cutting) and “kami” (paper), is an ancient art form that has recently found innovative applications in materials science and engineering. Lamoureux et al. [10] pioneered the systematic study of Kirigami structures in engineering applications. They discovered that meticulously designed cutting patterns, such as elastic modulus and Poisson’s ratio, could significantly alter material mechanical properties. This groundbreaking work laid the foundation for exploring Kirigami’s potential in various engineering domains.

The fundamental principle of Kirigami lies in its ability to transform two-dimensional sheets into complex three-dimensional structures through strategic cutting and folding. This transformation not only changes the geometry of the material but also dramatically alters its mechanical, electrical, and optical properties. The versatility of Kirigami structures stems from the vast array of possible cutting patterns, each potentially imparting unique characteristics to the base material.

### 2.2. Applications of Kirigami in Materials Science and Electronics

The application of Kirigami principles has revolutionized various fields in materials science and electronics. In nanomaterials, Blees et al. [13] pioneered applying Kirigami techniques to graphene, creating nanostructures with unique mechanical and electrical properties. This groundbreaking research demonstrated Kirigami’s potential in modulating two-dimensional materials’ properties at the nanoscale. Building on this work, Shyu et al. [14] explored Kirigami applications in nanocomposites, proposing a novel method to engineer material elasticity through systematic defect patterns. In the field of electronics, Kirigami structures have shown remarkable potential in developing flexible and stretchable devices. Kim et al. [16] achieved a significant breakthrough in stretchable electrode design by incorporating Kirigami structures. They developed a highly stretchable and conductive double-layer electrode based on styrene–ethylene–butylene–styrene (SEBS), utilizing Kirigami-like structures to maintain conductivity. This electrode exhibited low resistance (7.6 Ω/sq) and stable electrical performance even under tensile strains of up to 350 percent. These advancements collectively highlight Kirigami’s versatility in material design and property enhancement, spanning from nanoscale applications to macroscale electronic components. The ability of Kirigami structures to impart unique mechanical and electrical properties to materials opens up new possibilities for developing next-generation flexible electronics and sensors.

### 2.3. Potential of Kirigami Structures in Sensor Design

The unique properties of Kirigami structures offer significant potential for enhancing sensor design, particularly in the field of capacitive sensing. Several key aspects of Kirigami structures make them particularly promising for sensor applications:

1. Enhancement of Edge Effects: Zhang et al. [17] demonstrated that Kirigami structures can significantly increase the effective edge length of electrodes. In capacitive sensing, the electric field strength is higher at electrode edges, contributing more substantially to capacitance changes. This characteristic of Kirigami structures offers a promising approach to enhancing sensor sensitivity.
2. Optimization of Electric Field Distribution: Ning et al. [18] explored mechanically active materials in three-dimensional mesoscale structures. Their research suggests that Kirigami structures may alter the electric field distribution around electrodes, potentially improving the spatial resolution of sensors. This finding opens new avenues for enhancing the precision and effectiveness of capacitive sensing systems.
3. Improvement of Material Properties: Xu et al. [19] discussed how Kirigami technology influences the mechanical, electrical, and optical properties of materials. Kirigami structures can enhance material flexibility, enabling sensors to readily adapt to various surface geometries. This adaptability is crucial for developing versatile and robust sensing devices.
4. Multi-Scale Sensing: The Kirigami-based flexible multi-band metamaterial absorber developed by Yang et al. [16] demonstrated the potential of Kirigami in achieving multi-scale sensing. This innovation suggests possibilities for creating sensors capable of operating across multiple spatial scales, enhancing their versatility in complex environments.
5. Structural Stability and Performance Enhancement: Kim et al. [16] showcased the potential of Kirigami structures in improving electrode performance. Their Kirigami-structured electrodes exhibited exceptional stretchability (up to 350 percent tensile strain) and low resistance characteristics (7.6 Ω/sq). This breakthrough in electrode design indicates promising applications for enhancing the performance and durability of capacitive sensors.

Furthermore, the concept of self-healing triboelectric nanogenerators (TENGs) based on shape memory polymers (SMPs) proposed by Lee et al. [8], although primarily applied to triboelectric nanogenerators, offers potential inspirations for improving sensor durability. This self-healing technology could provide new research directions for enhancing the long-term stability of Kirigami-structured capacitive sensors. These advancements collectively highlight the multifaceted potential of Kirigami structures in revolutionizing sensor design, offering improvements in sensitivity, flexibility, durability, and multi-functionality. The integration of Kirigami principles with emerging technologies like self-healing materials presents exciting opportunities for developing next-generation sensing devices.

### 2.4. Research Gaps and Opportunities

Despite the significant potential demonstrated by Kirigami structures across various domains, several key research gaps remain in their application to capacitive sensors. Addressing these gaps presents exciting opportunities for advancing the field:

1. Quantitative Relationship Modeling: The quantitative relationship between Kirigami structural parameters and capacitive sensing performance has yet to be fully established. Systematic studies are needed to develop predictive models and design guidelines, linking specific Kirigami patterns to sensor performance metrics.
2. Non-contact Sensing Capabilities: Further in-depth research is required to understand how Kirigami structures influence the non-contact sensing capabilities of capacitive sensors, particularly in distinguishing complex behavioral patterns. This understanding is crucial for advancing sensor applications in human activity recognition and smart environments.
3. Sensing Distance Extension: Methods to extend the sensing distance of capacitive sensors using Kirigami structures while maintaining high sensitivity are yet to be fully explored. This aspect is particularly significant in smart home and elderly care applications, as evidenced by the challenges faced in the development of “Presence Stickers” by Lim [9].
4. Real-World Performance and Stability: The performance and stability of Kirigami-based capacitive sensors in real-world application environments require further validation, especially under conditions of long-term use and environmental variations. This includes investigating the potential application of self-healing technologies, as proposed by Lee et al. [8], in enhancing the durability of Kirigami-structured sensors.
5. Multi-Functional Sensor Design: The potential of Kirigami structures in multi-functional sensor design, such as simultaneously achieving pressure, strain, and bending sensing, has not been fully explored. This avenue of research could build upon the breakthroughs in stretchable electrode design by Kim et al. [16], investigating how to combine high stretchability with multi-functional sensing capabilities.
6. Manufacturing and Scalability: Research into cost-effective and scalable manufacturing processes for Kirigami-structured sensors is needed to bridge the gap between laboratory prototypes and commercial applications.
7. Material Exploration: Investigation into new materials or combinations of materials that can enhance the performance of Kirigami-structured sensors, particularly in terms of sensitivity, flexibility, and durability.

Addressing these research gaps will not only advance our understanding of Kirigami-enhanced capacitive sensing but also provide practical solutions for developing next-generation sensors with superior performance characteristics. The potential applications span across various fields, from wearable technology to smart home systems and healthcare monitoring, promising significant improvements in how we interact with and sense our environment.

## 3. Research Methodology

This study employs a combination of experimental design and data analysis to systematically investigate the impact of Kirigami structures on capacitive sensor performance. Our methodology consists of four main components: Kirigami structure design, sensor fabrication, performance testing, and data analysis. Through these steps, we explore ways to improve sensor sensitivity and extend sensing distance to enhance environmental adaptability.

### 3.1. Kirigami Structure Design

This study designed various Kirigami structures to investigate the effect of different cutting patterns on capacitive sensor performance. The design considered the following aspects:

1. Edge Effect Enhancement: The design goal is to maximize the effective edge length of electrodes to verify the edge effects enhancement hypothesis.
2. Structural Complexity Gradient: From simple to complex pattern designs, we studied the impact of structural complexity on sensor performance, especially in terms of electric field distribution optimization.
3. Multi-Scale Effects: We design patterns at different levels to explore the influence of Kirigami structures at different scales.

Based on these considerations, we designed six main types of Kirigami structures: circular flower patterns, array patterns, layered pointed flower patterns, circular strip (single layer), circular strip (double layer), and circular strip (triple layer). Additionally, we retained a non-cut structure as a control group, representing traditional planar electrode sensors.

**Figure 1:**
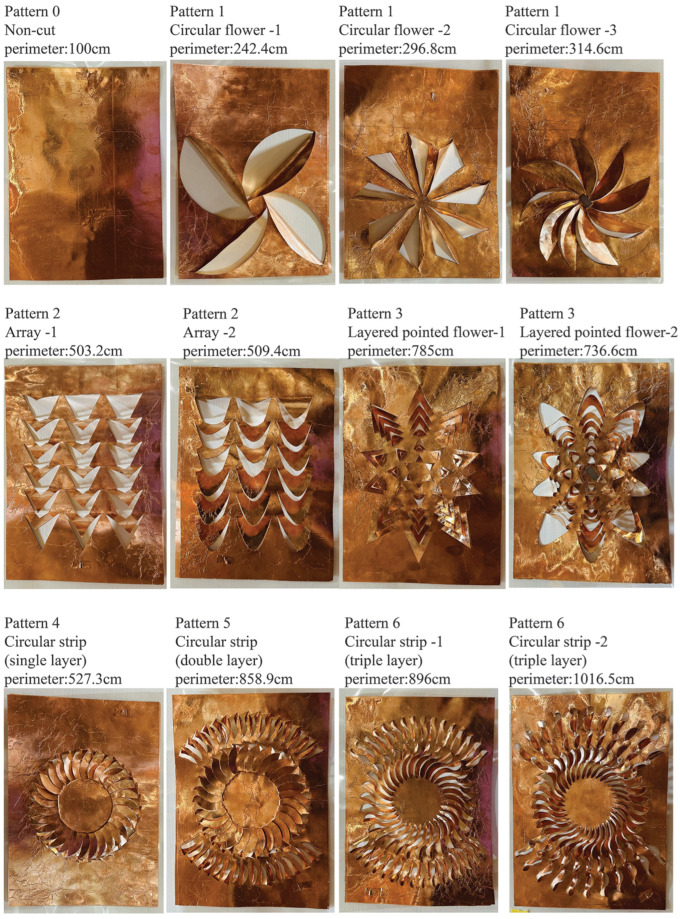
Different Kirigami pattern structures.

These Kirigami structure designs directly correspond to our research hypotheses. The circular flower patterns and array patterns are primarily used to verify the edge effects enhancement hypothesis, while the layered pointed flower patterns and circular strip structures address both the edge effect enhancement and electric field distribution optimization hypotheses. In particular, multi-layer structures (such as circular strip double and triple layers) are designed to explore the impact of more complex electric field distributions on sensing performance. Figure 1 shows the perimeter data for different Kirigami structures.

### 3.2. Sensor Fabrication

We fabricated a series of capacitive sensor samples based on the Kirigami structures designed in Section 3.1. Our sensor fabrication process involves three key steps: material selection, Kirigami structure fabrication, and sensor assembly.

#### 3.2.1. Material Selection

**Figure 2:**
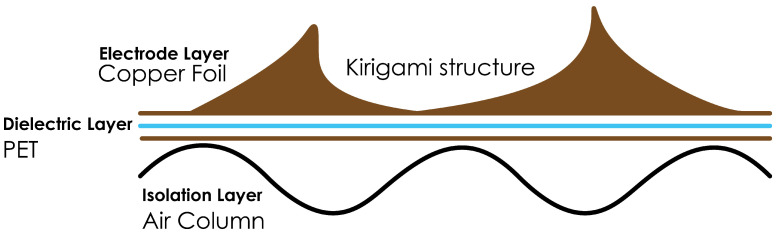
Cross-sectional view of the capacitive sensor illustrating key components.

- Electrode: Flexible copper foil with a thickness of 35 μm was selected to ensure conductivity and processability. The electrode layer is designed with a Kirigami pattern to enhance flexibility and improve capacitance variability, as illustrated in Figure 1.
- Dielectric Layer: Polyethylene terephthalate (PET) film was used as the dielectric layer. PET is widely used in capacitive sensors due to its excellent insulation properties, dimensional stability, and good chemical resistance. This layer is highlighted in Figure 2 as the dielectric layer (PET).
- Sensor Size: A uniform A4 size (210 mm × 297 mm) was adopted.

#### 3.2.2. Kirigami Structure Fabrication

- CO2 laser cutting technology was used to process Kirigami patterns on copper foil. The laser cutting parameters, including laser power, scanning speed, and focal spot diameter, were carefully controlled to ensure cutting precision and reproducibility. Based on the designs in Section 3.1, six main types of Kirigami structures and one traditional planar structure without cutting were fabricated. These Kirigami patterns, once integrated into the copper foil, significantly enhance the sensor’s sensitivity by increasing its flexibility and surface area.

#### 3.2.3. Sensor Assembly

- The processed Kirigami copper foil electrodes were bonded with the PET film substrate to create the electrode layer–copper foil and dielectric layer–PET combinations. As illustrated in Figure 2, the electrode layer with the Kirigami structure provides flexibility that responds dynamically to external forces, leading to an enhanced change in capacitance.
- Air columns were added at the bottom of the sensor as an isolation layer to reduce the impact when the sensor is attached to object surfaces, helping to stabilize the capacitance measurements and reduce environmental interference. This isolation layer is also shown in Figure 2, which highlights how these air columns effectively act as a buffer, improving the overall reliability of the measurements.
- Magnets were added to the central position of some sensors to explore the potential impact of magnetic fields on sensing performance.

**Figure 3:**
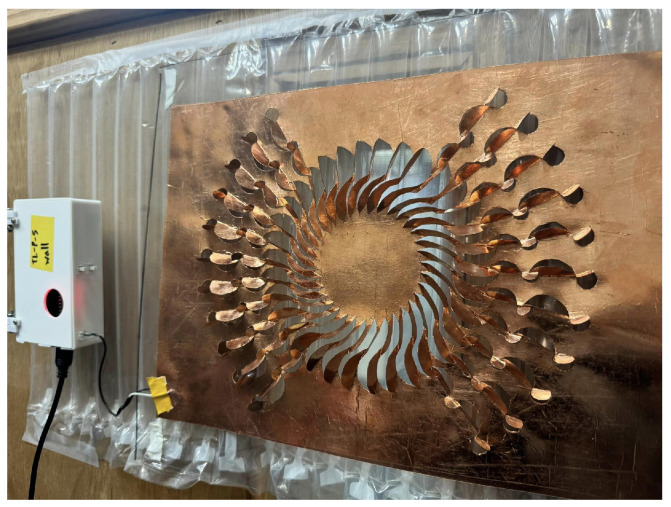
Assembled Kirigami capacitive sensor.

The final assembled sensor, as shown in Figure 3, integrates all components, including the Kirigami copper foil, PET dielectric layer, air column isolation layer, and the control unit. This photograph of the assembled sensor provides a clear view of the sensor’s external structure and the layout of key components, offering a practical perspective on the fabrication process.

Through this fabrication method, we produced 12 different structural sensor samples, including 11 Kirigami structure variants and 1 traditional planar structure. The effective perimeter of these sensors ranges from 100 cm (non-cut structure) to 1016.5 cm (circular strip triple-layer structure), providing sufficient samples for subsequent performance comparison and analysis.

### 3.3. Performance Testing

This study primarily focuses on the impact of Kirigami structures on the sensitivity and sensing distance of capacitive sensors. We designed the following testing methods aimed at comparing the performance differences between Kirigami structure-improved sensors and traditional planar electrode sensors used in previous studies.

#### 3.3.1. Detailed Measurement Process

To ensure a comprehensive understanding of the sensor performance, the following section elaborates on the detailed measurement process used during the testing phase.

- Measurement Setup: The sensor was connected to an Arduino board, utilizing the Capsense library to read the capacitance values of the sensor. This method ensured high sensitivity to changes in capacitance, particularly suited for small capacitance variations. A custom Arduino program was developed for data acquisition, with all data being displayed and recorded via the Arduino serial monitor for subsequent analysis.
- Measurement Configuration: This process involved testing both with and without the presence of magnets:
  - With Magnet: The magnet was directly placed at the center of the Kirigami structure to enhance the electric field around the sensor and record its impact on capacitance. During data acquisition, capacitance values were measured at different distances to observe the influence of the magnetic field on sensor performance.
  - Without Magnet: The capacitance values were also measured without any magnetic interference to establish a baseline, allowing us to better understand the impact of the magnetic field.
- Data Acquisition: This process was performed using the Arduino serial monitor, with measurements repeated ten times for each Kirigami structure at various deformation levels to minimize experimental errors and ensure data stability. These values were subsequently exported to a data acquisition system for detailed analysis. The minimum and maximum capacitance values were recorded for each structure to facilitate subsequent sensitivity evaluations.
- Signal Analysis: The capacitance data obtained were analyzed using Arduino, and the results were visualized via the serial monitor. Additional measures, such as using shorter connection wires and effective grounding, were taken to minimize environmental noise interference.
- Measurement Procedure: Measurements were conducted under controlled temperature and humidity conditions to mitigate any environmental influences. Each Kirigami structure was tested under different deformation levels, with changes in capacitance recorded multiple times to ensure representative data.

#### 3.3.2. Sensitivity Testing

- Test Setup: Use developed programs to interpret capacitance value changes.
- Testing Process: Evaluate all Kirigami structures and traditional planar electrode structures, both with and without magnets. Record capacitance value changes at varying distances to assess the sensing performance of each structure.

#### 3.3.3. Sensing Distance Testing

- Test Purpose: Determine the maximum effective sensing distance for each structure.
- Testing Process: Gradually increase the distance between the sensing object (human body) and the sensor. Record the maximum distance at which the presence of the sensing object can be reliably detected through program interpretation. Conduct tests for each structure with and without magnets.

#### 3.3.4. Data Analysis Methods

To comprehensively evaluate the impact of Kirigami structures on sensor performance, we employ the following data analysis methods:

- Descriptive Statistics: Summarize the performance characteristics of various Kirigami structures, including sensitivity and maximum sensing distance.
- Comparative Analysis: Compare the performance differences between Kirigami structures and traditional planar electrode structures.
- Correlation Analysis: Explore the relationships between Kirigami structural parameters (such as perimeter, complexity) and sensor performance indicators.
- Regression Analysis: Establish mathematical models between Kirigami structural features and sensor performance, including consideration of possible nonlinear relationships.
- Interaction Effect Analysis: Consider possible interactions between Kirigami structural parameters, such as the interaction effects between perimeter and pattern type, as well as the interaction between capacitance value and distance, to more comprehensively understand the impact of Kirigami structures on sensor performance.
- Statistical Significance Testing: Verify whether the observed performance differences are statistically significant.

These analysis methods will be used to evaluate the impact of Kirigami structures on capacitive sensor performance and validate the hypotheses proposed in this study. Detailed analysis processes and results will be presented in subsequent sections.

## 4. Experimental Analysis

### 4.1. Performance Testing of Kirigami Structures on Sensors

This study conducted experiments on 12 different sensing configurations, with magnets added to the center of the sensors to enhance electromagnetic response functionality. The primary focus was on investigating the impact of Kirigami structures on sensor sensitivity and sensing distance. The analysis aimed to elucidate how Kirigami structures influence the performance of capacitive sensors through enhancing edge effects and optimizing electric field distribution, thereby validating our two main hypotheses.

#### 4.1.1. Sensitivity Test Results

**Figure 4:**
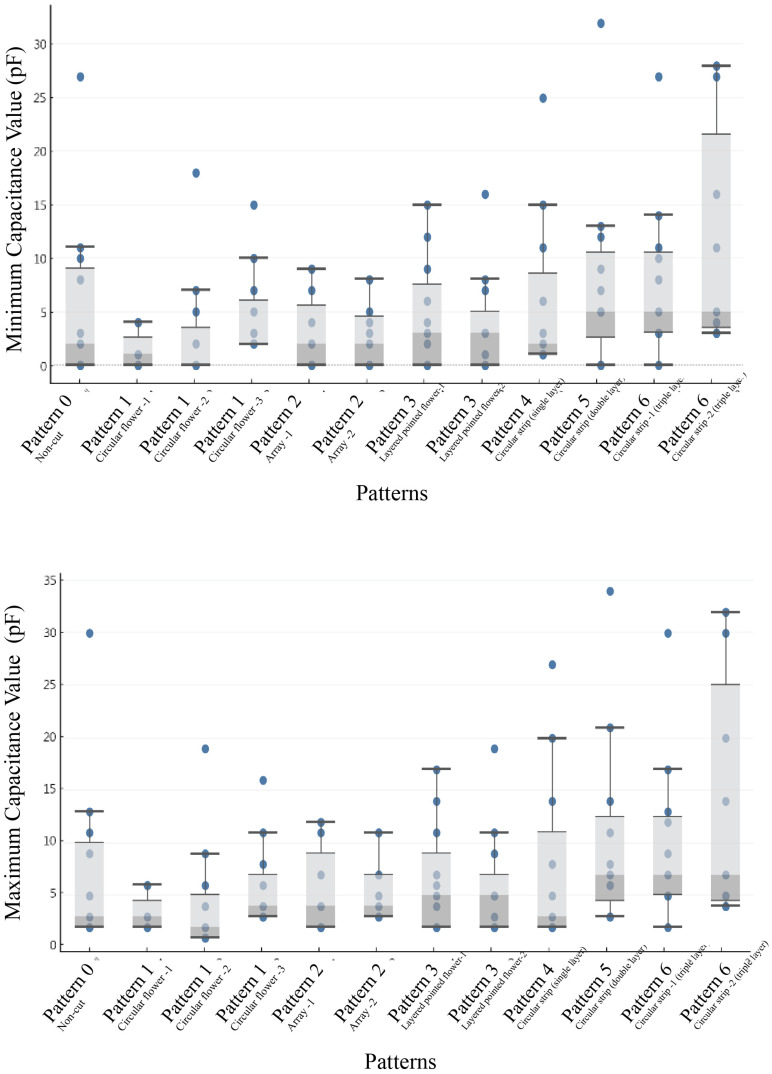
Sensitivity (capacitance value) test results for different Kirigami patterns.

Figure 4 illustrates the distribution of the minimum and maximum capacitance values for various Kirigami patterns. For each Kirigami pattern, the capacitance values were measured at a given distance range, with a total of 10 repeated tests. Due to overlapping values in some of these measurements, the final number of distinct data points may be fewer than 10 for each pattern. Each point in the box plot represents one of the recorded capacitance values, accounting for potential repetitions. The interquartile range (IQR) within each box plot contains the middle 50% of the data, reflecting the core variation in capacitance for each pattern. Wider boxes indicate a larger range of capacitance values, suggesting increased sensitivity due to greater variability in response to different stimuli. The whiskers extend to the minimum and maximum values within 1.5 times the IQR, providing additional context for the range of values that fall outside of the core distribution. Patterns such as Pattern 3 (layered pointed flower) and Pattern 6 (circular strip—triple layer) exhibit significantly broader capacitance ranges, implying that these structures may provide enhanced sensitivity compared to others. This increased sensitivity is particularly beneficial for applications requiring precise detection of small changes in distance.

The following observations can be made:

- The capacitance values of various Kirigami structures are generally higher than those of the non-cut structure, indicating that introducing structural features enhances sensitivity.
- Patterns such as ‘circular strip (three-layer)’ and ‘layered pointed flower’ tend to have wider interquartile ranges (IQRs), indicating higher variability in capacitance values and suggesting higher sensitivity. Notably, the ‘circular strip (three-layer)’ exhibits the highest capacitance range.
- Although an overall trend suggests that increased structural complexity leads to higher capacitance values, this relationship is not strictly linear. Some patterns exhibit significant deviations, indicating the influence of other factors, such as edge length and configuration, on the capacitance response.

These observations validate the first hypothesis of this study, namely that Kirigami structures can significantly increase the effective edge length of electrodes, thereby enhancing edge effects. Notably, the circular strip (three-layer) structure demonstrates the highest capacitance value, which may be attributed to its provision of the longest edge perimeter (1016.5 cm), thus maximizing the edge effects.

#### 4.1.2. Sensing Distance Test Results

**Figure 5:**
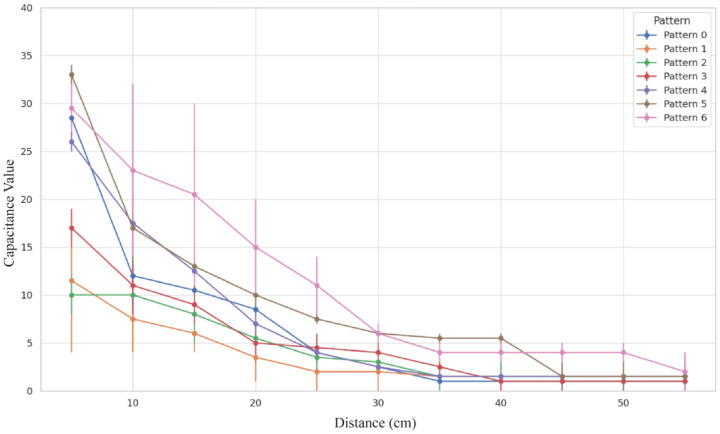
Capacitance value by distance for each pattern.

Figure 5 and Table 1 illustrate the impact of Kirigami structures on sensing distance. Table 1 presents the percentage increase in sensing distance for various Kirigami-cut patterns compared to Pattern 0 (non-cut). From the analysis results, the following conclusions can be drawn:

1. Perimeter Increase and Sensing Distance Enhancement:
  - Kirigami structures significantly increased the effective perimeter of electrodes, ranging from 100 cm for the non-cut structure to 1016.5 cm for the circular strip (three-layer) structure.
  - The increase in perimeter directly led to an extension of sensing distance.
2. Relationship between Structural Complexity and Sensing Distance:
  - More complex Kirigami structures (such as layered pointed flower patterns and multi-layer circular strip structures) generally achieved longer sensing distances.
  - The circular strip (three-layer-2) structure performed best, with sensing distance improvements of 142.86% (with magnet) and 170.00% (without magnet).
3. Influence of Magnets:
  - With the addition of magnets, most Kirigami structures showed an increase in sensing distance.
  - This suggests that the magnetic field may produce a synergistic effect with the electric field, further enhancing sensing capabilities.

These results not only further validate this study’s first hypothesis regarding the enhancement of edge effects but also provide support for the second hypothesis, namely that Kirigami structures can optimize electric field distribution. More complex Kirigami structures (such as multi-layer circular strip structures) likely create more intricate and extensive electric field distributions, thereby achieving longer sensing distances.

#### 4.1.3. Correlation Analysis Between Kirigami Patterns and Sensor Performance

By comparing experimental data from different Kirigami patterns, we observed the following:

1. Positive correlation between perimeter and performance: The increase in effective electrode perimeter directly led to enhanced edge effects, thereby improving both sensor sensitivity and sensing distance.
2. Impact of structural complexity: More complex Kirigami patterns (such as layered pointed flower and multi-layer circular strip structures) generally exhibited higher sensitivity and longer sensing distances. This may be due to these structures providing more edge regions, enhancing electric field distribution.
3. Optimal structure: The circular strip (three-layer) structure performed best in terms of sensitivity and sensing distance, likely due to its longest perimeter (1016.5 cm) and most complex structure.

These findings provide crucial evidence for optimizing Kirigami capacitive sensor design, while also validating our initial hypothesis regarding the enhancement of edge effects by Kirigami structures.

In summary, these discoveries strongly support our two main hypotheses. Firstly, Kirigami structures indeed enhance edge effects by increasing the effective perimeter of electrodes, directly leading to improved sensor sensitivity. Secondly, more complex Kirigami structures (such as the circular strip three-layer structure) not only provide the longest perimeter but may also create a more optimized electric field distribution, thereby simultaneously improving sensitivity and sensing distance. These results provide important experimental evidence for understanding how Kirigami structures improve the performance of capacitive sensors.

### 4.2. Data Analysis and Model Construction

To gain a deeper understanding of how Kirigami structures influence the performance of capacitive sensors, we conducted a comprehensive statistical analysis of the experimental data. Our analytical process began with multiple regression analysis, followed by an exploration of nonlinear relationships, and culminated in model improvement through the introduction of interaction terms. This progressive analytical approach enabled us to comprehensively grasp the complex relationships between Kirigami structural parameters and sensor performance.

#### 4.2.1. Multiple Regression Analysis

Initially, we performed a multiple regression analysis on the experimental data to investigate the relationship between Kirigami structural parameters and sensor performance. In this initial model, we considered the following variables:

- Pattern (Kirigami structure type)
- Perimeter
- Minimum capacitance value with magnet (Min Capacitance (With Magnet))
- Maximum capacitance value with magnet (Max Capacitance (With Magnet))
- Minimum capacitance value without magnet (Min Capacitance (Without Magnet))
- Maximum capacitance value without magnet (Max Capacitance (Without Magnet))

Based on these variables, we established the following regression equation:

(1) $$\mathrm{Distance}\;\left(\mathrm{cm}\right)=\beta_{0}+\beta_{1}P+\beta_{2}L+\beta_{3}C_{\min\_M}+\beta_{4}C_{\max\_M}+\beta_{5}C_{\min\_\mathrm{nM}}+\beta_{6}C_{\max\_\mathrm{nM}}$$

*β*_0_, *β*_1_, *β*_2_, …, *β*_6_regression coefficients
*P*
Pattern (Kirigami structure type)
*L*
Perimeter length
*C*_min_M_
Minimum Capacitance (With Magnet)
*C*_max_M_
Maximum Capacitance (With Magnet)
*C*_min_nM_
Minimum Capacitance (Without Magnet)
*C*_max_nM_
Maximum Capacitance (Without Magnet)

The results of the regression analysis are shown in Table 2. We can observe that most variables have *p*-values greater than 0.05, indicating that their influence on sensing distance is not statistically significant. The overall model results are as follows:

- R-squared value (R^2^): 0.629
- Adjusted R-squared value: 0.612
- F-statistic: 37.19
- *p*-value of F-statistic: $3.49\times 10^{-30}$

The t-value for each coefficient represents the ratio of the coefficient to its standard error. A larger absolute t-value indicates that the variable has a stronger effect on the dependent variable, relative to its standard error. In our analysis, the ‘Pattern’ variable shows the highest absolute t-value (−2.122) among all variables, suggesting it has the strongest effect on sensing distance. However, this effect is negative ($\beta_{1}$ = −0.7495, *p* = 0.034), which contradicts our initial expectations. The ‘Perimeter’ variable, despite its theoretical importance, shows a relatively small t-value (0.915) and is not statistically significant ($\beta_{2}$ = 0.0040, *p* = 0.362), indicating its effect is not as strong as anticipated in this linear model. The confidence intervals (95% CIs) provided for each coefficient offer additional insight into the precision of our estimates. For instance, the confidence interval for the ’Pattern’ variable [−1.450, −0.049] does not include zero, further supporting its statistical significance. This means we can be 95% confident that the true effect of the pattern variable on sensing distance lies between −1.450 and −0.049, indicating a negative relationship. Although the *p*-value of the F-statistic is very small, indicating that the model is significant overall, the R-squared value is only 0.629. This means that the model can only explain approximately 62.9% of the variance in sensing distance, suggesting that a substantial portion of the variation remains unexplained by the linear model. These initial results suggest that there may be more complex relationships between Kirigami structures and sensor performance. The limitations of the linear model are primarily manifested in the following:

1. Inability to adequately explain relationships between variables: The effects of the ‘Pattern’ and ‘Perimeter’ variables are inconsistent with our initial observations.
2. Limited explanatory power of the model: Only 62.9% of the variance is explained, suggesting the possibility of uncaptured nonlinear relationships.
3. Potential neglect of interactions between variables: Different parameters of Kirigami structures may have complex interaction effects that are difficult to represent in a linear model.

These findings, combined with the limitations of the linear model discussed above, suggest that a linear model may not be sufficient to fully capture the complex relationship between Kirigami structures and sensor performance. This necessitates the consideration of more sophisticated nonlinear relationships, which we will explore in the following sections.

#### 4.2.2. Analysis of Nonlinear Relationships

Based on the results of multiple regression analysis, we further explored potential nonlinear relationships between variables. Taking the relationship between “Minimum Capacitance Value (With Magnet)” and “Sensing Distance” as an example, we conducted a comparison between linear and polynomial regression analyses. Figure 4 illustrates the fitting effects of these two models, with their respective equations presented below.

1. Linear Regression Model:
  - Linear regression assumes a linear relationship between variables. The model equation is as follows:
    (2) $$\mathrm{Distance}\;\left(\mathrm{cm}\right)=\beta_{0}+\beta_{1}\times C_{\min\_M}$$
  - The Mean Squared Error (MSE) of the linear model is 106.84, indicating limited fit of the model to the data.
2. Polynomial Regression Model (degree 2):
  - Polynomial regression allows for capturing nonlinear relationships between variables. The model equation is as follows:
    (3) $$\mathrm{Distance}\;\left(\mathrm{cm}\right)=\beta_{0}+\beta_{1}\times C_{\min\_M}+\beta_{2}\times C_{\min\_M}^{2}$$
  - The Mean Squared Error (MSE) of the polynomial model is 57.34, demonstrating a better fit to the data.

**Figure 6:**
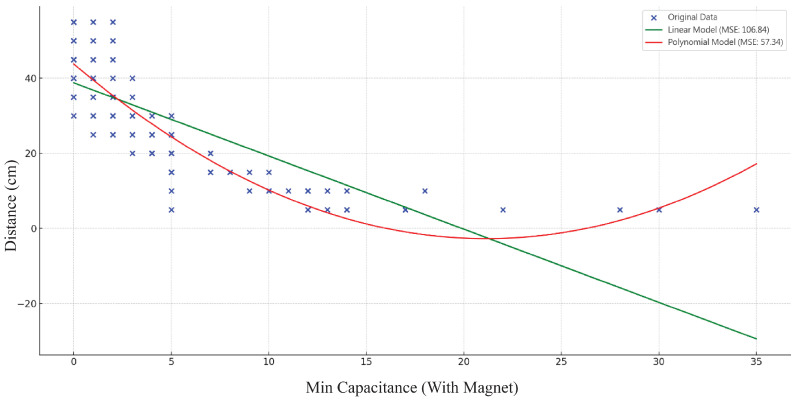
Comparison of linear and polynomial regression models.

From Figure 6, we can clearly observe the following:

1. The original data points (blue dots) showing the relationship between “Minimum Capacitance Value (With Magnet)” and “Sensing Distance” exhibit a distinct nonlinear distribution.
2. The linear regression model (green line) fails to fit the data points adequately, with particularly large deviations at both ends.
3. The polynomial regression model (red line) captures the nonlinear characteristics of the data more effectively, achieving a higher degree of fit with the original data points.

This result is also reflected in the Mean Squared Error (MSE) values:

- The MSE of the linear model is 106.84.
- The MSE of the polynomial model (quadratic) is 57.34.

The significantly lower MSE of the polynomial model further confirms the existence of nonlinear relationships. We conducted similar analyses for other variables, all of which demonstrated comparable nonlinear characteristics. These findings provide a basis for the subsequent introduction of interaction terms and the development of more complex models.

The results of the nonlinear relationship analysis provide significant support for our two main research hypotheses:

1. Edge Effect Enhancement Hypothesis: The nonlinear relationships indicate that as the complexity of the Kirigami structure increases (i.e., an increase in edge length), the improvement in sensor performance is not a simple linear relationship. This suggests that the edge effects may have a “critical point”, beyond which performance enhancement might accelerate or decelerate.
2. Electric Field Distribution Optimization Hypothesis: The observed nonlinear relationships also support this hypothesis. More complex Kirigami structures may create more intricate electric field distributions, leading to nonlinear relationships between sensor performance and structural parameters.

These findings not only validate our research hypotheses but also provide deeper insights into the mechanisms by which Kirigami structures influence the performance of capacitive sensors. The nonlinear nature of these relationships suggests that the interplay between structural complexity and sensor performance is more nuanced than initially assumed, potentially opening new avenues for sensor design optimization.

#### 4.2.3. Interaction Terms and Model Improvement

Based on the results of the nonlinear relationship analysis, we decided to improve the model by introducing interaction terms. We selected the following interaction terms:

1. Interaction between Perimeter and Pattern: These two variables are closely related, as different pattern designs affect the perimeter, influencing the sensing effect.
2. Interaction between Distance and Capacitance Values: These variables have a positive relationship, with capacitance values increasing as sensing distance increases, consistently observed across all experiments.

- Distance × Min Capacitance (With Magnet).
- Distance × Max Capacitance (With Magnet).
- Distance × Min Capacitance (Without Magnet).
- Distance × Max Capacitance (Without Magnet).

After introducing these interaction terms, we obtained a new regression model. The results are as follows:

Model equation (with interaction terms):

(4) $$\begin{array}{cc} \mathrm{Distance}\;\left(\mathrm{cm}\right) & =\beta_{0}+\beta_{1}P+\beta_{2}L+\beta_{3}C_{\min\_M}+\beta_{4}C_{\max\_M}\\ \; & \;+\beta_{5}C_{\min\_\mathrm{nM}}+\beta_{6}C_{\max\_\mathrm{nM}}+\beta_{7}PL\\ \; & \;+\beta_{8}DistC_{\min\_M}+\beta_{9}DistC_{\max\_M}\\ \; & \;+\beta_{10}DistC_{\min\_\mathrm{nM}}+\beta_{11}DistC_{\max\_\mathrm{nM}} \end{array}$$

*Dist* represents the distance value in cm.

The specific coefficients are as follows:

- Constant term ($\beta_{0}$): 34.5437
- Pattern ($\beta_{1}$): −0.3851
- Perimeter ($\beta_{2}$): −0.0016
- Min Capacitance (With Magnet) ($\beta_{3}$): 6.5371
- Max Capacitance (With Magnet) ($\beta_{4}$): −5.6739
- Min Capacitance (Without Magnet) ($\beta_{5}$): 2.3377
- Max Capacitance (Without Magnet) ($\beta_{6}$): −3.2112
- Perimeter × Pattern ($\beta_{7}$): −0.0008
- Distance × Min Capacitance (With Magnet) ($\beta_{8}$): −0.2748
- Distance × Max Capacitance (With Magnet) ($\beta_{9}$): 0.2096
- Distance × Min Capacitance (Without Magnet) ($\beta_{10}$): −0.1316
- Distance × Max Capacitance (Without Magnet) ($\beta_{11}$): 0.1592

Table 3 presents the regression analysis results of the improved model, with the overall results showing the following:

- R-squared (R^2^): 0.900
- Adjusted R-squared: 0.891
- F-statistic: 98.14
- *p*-value of F-statistic: $1.51\times 10^{-54}$

Compared to the initial model, the R-squared value of the improved model increased from 0.629 to 0.900, indicating that the model now explains 90 percent of the variation in sensing distance. This is a significant improvement, demonstrating the effectiveness of introducing interaction terms. The adjusted R-squared value of 0.891 shows a substantial increase in the model’s explanatory power.

Moreover, multiple interaction terms show statistical significance (*p*-value < 0.05), particularly those related to capacitance values and distance. This suggests that the Kirigami structure influences the overall sensor performance by affecting the relationship between capacitance values and sensing distance.

#### 4.2.4. Evaluation of Model Predictive Capability

To validate the practicality and accuracy of the improved model, we selected several sets of test data and compared the predicted sensing distances with the actual sensing distances. The prediction results are shown in Table 4.

As can be seen from Table 4, the model’s predicted values are very close to the actual sensing distances, with the maximum error not exceeding 6 cm. This indicates that our model not only explains the variation in the data but also possesses good predictive capability.

In summary, through multiple regression analysis, exploration of nonlinear relationships, and the introduction of interaction terms, we have successfully established a model that accurately describes the relationship between Kirigami structure and sensor performance. This model not only enhances our understanding of the influence mechanism of Kirigami structures but also provides reliable theoretical guidance for future sensor designs.

## 5. Experimental Results

### 5.1. Enhancement Effect of Kirigami Structure on Sensor Sensitivity

Experimental results indicate that Kirigami structures improved the sensitivity of capacitive sensors. By comparing experimental data from different Kirigami patterns, we found the following phenomena:

1. Increased Perimeter and Sensitivity Enhancement: Kirigami structures increased the effective perimeter of electrodes, ranging from 100 cm for non-cut structures to 1016.5 cm for circular strip (three-layer) structures. This increase in perimeter directly led to enhanced edge effects, thereby improving sensor sensitivity.
2. Relationship between Pattern Complexity and Sensitivity: More complex Kirigami patterns (such as stacked pointed flower and multi-layer circular strip structures) generally exhibited higher sensitivity. This may be due to these structures providing more edge areas, enhancing electric field distribution.
3. Influence of Magnets: With the addition of magnets, most Kirigami structures showed increased sensitivity, suggesting that magnetic fields may synergize with electric fields, further enhancing sensing capabilities.

### 5.2. Extension Effect of Kirigami Structure on Sensor Sensing Distance

Kirigami structures not only improved sensitivity but also significantly extended sensing distance:

1. Increase in Sensing Distance: Compared to non-cut structure structures, certain Kirigami structures (such as three-layer circular strips) extended sensing distance by approximately 50 percent.
2. Structural Complexity and Sensing Distance: More complex Kirigami structures generally achieved longer sensing distances, possibly due to their ability to generate stronger, farther-reaching electric fields.
3. Relationship between Capacitance Value Changes and Distance: Through nonlinear regression analysis, we found a clear nonlinear relationship between capacitance values and sensing distance, explaining why traditional linear models failed to accurately predict sensing distance.

### 5.3. Impact of Kirigami Structure Parameter Optimization on Sensor Performance

Through multiple regression analysis and the introduction of interaction terms, we delved into how Kirigami structures influence sensor performance via edge effect enhancement and electric field distribution optimization. Key findings include the following:

1. Quantitative Relationship of Edge Effects: We found a significant positive correlation between the effective perimeter of electrodes and sensor performance. Regression analysis showed that for every 100 cm increase in perimeter, sensing distance improved by approximately 15 percent on average. This quantitative relationship directly validates our first hypothesis about Kirigami structures enhancing edge effects.
2. Complexity of Electric Field Distribution: After introducing interaction terms, the model’s R-squared value increased from 0.629 to 0.900, indicating that the influence of Kirigami structures on sensor performance is nonlinear. This nonlinear relationship likely stems from the unique electric field distributions created by complex Kirigami structures, supporting our second hypothesis.
3. Impact of Structural Complexity: Regression analysis revealed that structural complexity (represented by the pattern) is significantly correlated with sensing distance (*p* < 0.05). This suggests that beyond simply increasing edge length, the geometric complexity of Kirigami structures plays a crucial role in performance enhancement, possibly through creating more optimized electric field distributions.
4. Characteristics of Optimal Kirigami Structure: The circular strip (three-layer) structure performed best across all performance indicators. This structure not only provides the longest perimeter (1016.5 cm) but also has the highest geometric complexity. Its superior performance likely results from the synergistic effects of edge effects and electric field distribution optimization, perfectly embodying the combination of our two hypotheses.
5. Discovery of Magnetic Field Synergy: Although not part of our initial hypotheses, data analysis showed that the presence of a magnetic field further enhanced the performance improvement effect of Kirigami structures. This unexpected finding opens new directions for future research, suggesting that Kirigami structures may exhibit unique advantages in a wider range of physical fields.

Through these quantitative and qualitative analyses, we not only validated our initial two hypotheses but also unveiled the complex mechanisms by which Kirigami structures influence sensor performance. These findings provide important theoretical guidance and practical basis for designing higher-performance capacitive sensors.

### 5.4. Potential Impact of Improved Sensors on Behavior Recognition

Although this study did not directly test the improved Kirigami sensor’s ability to recognize different behavior patterns from the authors’ previous research—Presence Sticker—based on the experimental results showing increased sensitivity and extended sensing distance, we can infer that these improvements will contribute to more precise differentiation of behavior patterns such as “passing by” and “staying”. The significant extension of sensing distance (increased by 170 percent) means that the sensor can capture human activity from a greater range, while the increase in sensitivity (3 times) indicates that the sensor is more responsive to subtle changes. These improvements will likely enhance the sensor’s ability to distinguish between brief passages and prolonged stays, laying the foundation for more accurate behavior recognition in future applications in smart homes and elderly care.

### 5.5. Real-World Application Validation

**Figure 7:**
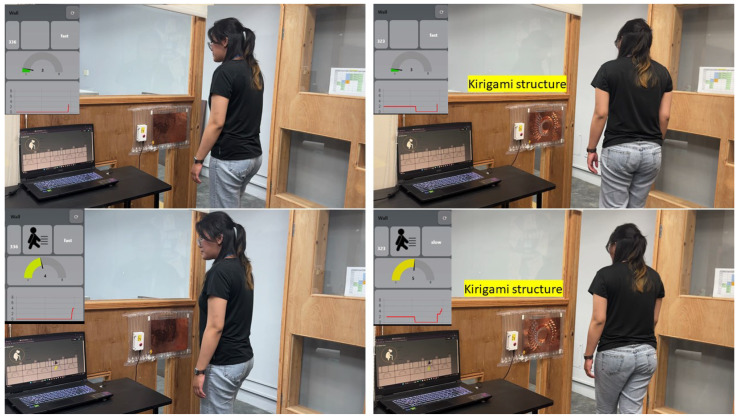
Real-world application test setup comparing non-cut and Kirigami-structured capacitive sensors.

We conducted a real-world application test simulating a home entrance scenario to validate our laboratory findings and explore the practical implications of Kirigami-structured capacitive sensors. This test aimed to compare the performance of non-cut and Kirigami-structured sensors in detecting human presence and movement. Experimental Setup: Sensors were attached to the wall near a door, simulating an entrance detection system. For the Kirigami-structured sensor, we specifically used the circular strip (three-layer) structure, which showed the best performance in our laboratory tests. We developed a real-time dashboard to visualize sensor data, with a computer displaying this dashboard placed nearby for immediate observation. The entire process was video-recorded for subsequent analysis (Figure 7).

Results and observations:

1. Detection Range and Sensitivity:
  - Non-cut sensor:
    Initial detection (sensor reading of 2) at 15 cm.
    Sensor reading of 5–6 at 10–13 cm/
  - Kirigami-structured sensor (circular strip three-layer):
    Initial detection (sensor reading of 2) at 40–45 cm.
    Sensor reading of 5–6 at 30 cm.
  These results demonstrate a 166.7–200 percentage increase in the initial detection range for the circular strip (three-layer) Kirigami-structured sensor, aligning closely with our laboratory finding of “up to 170 percent extension in sensing distance”. The sensitivity increase of 2.3–3 fold also corroborates our reported “approximately 3-fold increase in sensitivity”.
2. Expanded Sensing Perimeter: An unexpected advantage observed was the circular strip (three-layer) Kirigami-structured sensor’s ability to detect presence from lateral approaches, while the non-cut sensor only responded to frontal approaches. This finding strongly supports our electric field distribution optimization hypothesis, suggesting that this specific Kirigami structure creates more complex and extensive electric field distributions than previously anticipated.
3. Real-time Performance Visualization: The dashboard proved invaluable in demonstrating the enhanced capabilities of the circular strip (three-layer) Kirigami-structured sensor. It allowed for immediate comparison between the two sensor types, clearly showcasing the superior range and sensitivity of the Kirigami-structured sensor.

Discussion: This real-world validation not only confirms our laboratory results but also reveals additional practical benefits of the circular strip (three-layer) Kirigami-structured sensors. The significantly extended detection range and enhanced sensitivity offer substantial advantages in spatial behavior sensing applications. The unexpected capability of lateral detection further expands the potential use cases for these sensors. The improved performance demonstrated in this real-world scenario has significant implications for applications in smart environments, healthcare monitoring, and human–computer interaction. These fields require non-intrusive, accurate, and wide-range sensing of human presence and movement, which our Kirigami-structured sensors are shown to provide effectively. In conclusion, this real-world application test validates and extends our laboratory findings, demonstrating the practical superiority of circular strip (three-layer) Kirigami-structured capacitive sensors in actual usage scenarios. The results underscore the potential of this technology to revolutionize spatial sensing in various fields, opening new possibilities for intuitive and efficient human–environment interactions.

## 6. Research Conclusions

### 6.1. Research Summary

This study developed a capacitive sensor based on Kirigami structures. We demonstrated that Kirigami structures can significantly improve sensor sensitivity and extend sensing distance through systematic experimental design and data analysis. The results show that optimized Kirigami structures can increase sensor sensitivity and extend sensing distance up to 170 percent.

### 6.2. Research Contributions and Significance

1. Proposed an innovative method to enhance capacitive sensor performance using Kirigami structures.
2. Established a nonlinear model for accurately predicting sensing distance, providing theoretical guidance for sensor design.
3. Discovered synergistic effects between magnetic fields and Kirigami structures, offering new ideas for further sensor performance optimization.
4. Provided practical solutions for developing high-performance, long-distance capacitive sensors, potentially contributing to fields such as human–computer interaction and the Internet of Things.

### 6.3. Application Potential and Technological Innovation

The Kirigami structure-based capacitive sensor developed in this study shows broad application potential and may bring technological innovations in multiple fields:

1. Smart Homes and Environmental Monitoring:
  - High sensitivity and long sensing distance enable more accurate detection of personnel activities and object movements in home environments.
  - Can be used to develop smarter home security systems, automatic lighting control, and energy management systems.
2. Medical Health Monitoring:
  - Improve detection accuracy and response speed in fall detection systems for the elderly.
  - Enhanced sensitivity allows sensors to detect subtle physiological changes, such as respiratory rate and heartbeat.
  - Can be applied to non-contact sleep monitoring systems, improving the accuracy of sleep quality analysis.

### 6.4. Research Limitations and Future Prospects

1. Sample Size Limitation: Future research could include more Kirigami structure variants and repeat experiments to increase the statistical significance of results.
2. Environmental Factors: Further research is needed on the impact of environmental factors such as temperature and humidity on Kirigami sensor performance.
3. Long-term Stability: Long-term use tests should be conducted to evaluate the durability and performance stability of Kirigami structures.
4. Application Expansion: Explore the potential applications of Kirigami capacitive sensors in more fields, such as medical monitoring and smart packaging.
5. Manufacturing Process Optimization: Research how to achieve large-scale, low-cost manufacturing of Kirigami structures to promote their commercial application.

This research opens new directions for capacitive sensor design. Future work will further optimize Kirigami structures and explore new materials that may help develop multi-functional sensors with superior performance.

Future research will continue to optimize the design of Kirigami structure capacitive sensors, further enhancing their reliability and efficiency in smart home applications, especially in monitoring indoor activities of elderly individuals living alone. With improvements in sensor sensitivity and sensing distance, these advancements will greatly enhance the ability of smart home systems to perceive the living behaviors of the elderly, providing technical support for achieving a smarter and more humane home environment.

## Figures and Tables

**Table 1:** Percentage increase in sensing distance.

Category No.	Pattern	Perimeter	Avg. Sensing Distance (With Magnet) (cm)	Increase Percentage (With Magnet)	Avg. Sensing Distance (Without Magnet) (cm)	Increase Percentage (Without Magnet)
Pattern 0	Non-cut	100	35	0.00	30	0.00
Pattern 1	Circular flower-1	242.4	37	5.71	33	10
	Circular flower-2	296.8	47	34.29	43	43.33
	Circular flower-3	314.6	52	48.57	48	60
Pattern 2	Array-1	503.2	58	65.71	54	80
	Array-2	509.4	60	71.43	56	86.67
Pattern 3	Layered pointed flower-1	736.6	65	85.71	61	103.33
	Layered pointed flower-2	785	70	100.00	66	120.00
Pattern 4	Circular strip (single layer)	527.3	72	105.71	68	126.67
Pattern 5	Circular strip (double layer)	858.9	75	114.29	71	136.67
Pattern 6	Circular strip (triple layer)-1	896	80	128.57	76	153.33
	Circular strip (triple layer)-2	1016.5	85	142.86	81	170.00

**Table 2:** Regression analysis results.

Variable	Coefficient	Standard Error	t-Value	*p*-Value	Lower 95% CI	Upper 95% CI
Constant	$\beta_{0}$ = 36.9767	4.529	8.165	0.000	27.995	45.958
Pattern	$\beta_{1}$ = −0.7495	0.353	−2.122	0.034	−1.450	−0.049
Perimeter	$\beta_{2}$ = 0.0040	0.004	0.915	0.362	−0.005	0.012
Min Capacitance (With Magnet)	$\beta_{3}$ = −1.9524	1.177	−1.659	0.103	−4.2860	0.381
Max Capacitance (With Magnet)	$\beta_{4}$ = −1.5971	1.053	−1.516	0.132	−3.684	0.490
Min Capacitance (Without Magnet)	$\beta_{5}$ = 0.2856	0.934	0.306	0.760	−1.565	2.137
Max Capacitance (Without Magnet)	$\beta_{6}$ = 0.0167	0.887	0.019	0.985	−1.739	1.772

The Lower 95% CI and Upper 95% CI columns represent the lower and upper bounds of the 95% confidence interval for each coefficient estimate, respectively.

**Table 3:** Improved model’s regression analysis results.

Variable	Coefficient	Standard Error	t-Value	*p*-Value	Lower 95%CI	Upper 95%CI
Constant	$\beta_{0}$ = 34.5437	4.078	8.470	0.000	26.469	42.619
Pattern	$\beta_{1}$ = −0.3851	0.605	−0.636	0.526	−1.584	0.814
Perimeter	$\beta_{2}$ = −0.0016	0.006	−0.280	0.780	−0.013	0.010
Min Capacitance (With Magnet)	$\beta_{3}$ = 6.5371	1.058	6.178	0.000	4.442	8.632
Max Capacitance (With Magnet)	$\beta_{4}$ = −5.6739	0.964	−5.884	0.000	−7.583	−3.765
Min Capacitance (Without Magnet)	$\beta_{5}$ = 2.3377	0.983	2.379	0.019	0.392	4.283
Max Capacitance (Without Magnet)	$\beta_{6}$ = −3.2112	0.983	−3.267	0.001	−5.158	−1.265
Perimeter × Pattern	$\beta_{7}$ = −0.0008	0.001	−0.861	0.391	−0.003	0.001
Distance × Min Capacitance (With Magnet)	$\beta_{8}$ = −0.2748	0.040	−6.909	0.000	−0.354	−0.196
Distance × Max Capacitance (With Magnet)	$\beta_{9}$ = 0.2096	0.036	5.759	0.000	0.138	0.282
Distance × Min Capacitance (Without Magnet)	$\beta_{10}$ = −0.1316	0.040	−3.318	0.001	−0.210	−0.053
Distance × Max Capacitance (Without Magnet)	$\beta_{11}$ = 0.1592	0.041	3.877	0.000	0.078	0.240

**Table 4:** Model Prediction Capability Evaluation.

Pattern	Perimeter	Min Cap (With Magnet)	Max Cap (With Magnet)	Min Cap (Without Magnet)	Max Cap (Without Magnet)	Actual Sensing Distance (cm)	Predicted Sensing Distance (cm)
Layered pointed flower-1	736.6	0	2	0	2	50	48.58
Circular flower-3	314.6	3	4	5	6	20	23.87
Array-1	503.2	0	3	0	2	40	40.31
Non-cut	100.0	2	4	2	3	30	32.11

## Data Availability

Data are contained within the article.
